# Biomass and Neutral Lipid Production in Geothermal Microalgal Consortia

**DOI:** 10.3389/fbioe.2014.00082

**Published:** 2015-02-16

**Authors:** Kathryn F. Bywaters, Christian H. Fritsen

**Affiliations:** ^1^Division of Earth and Ecosystem Sciences, Desert Research Institute, Reno, NV, USA; ^2^Graduate Program of Environmental Science, University of Nevada Reno, Reno, NV, USA

**Keywords:** algae, geothermal, biofuel, energy, biomass

## Abstract

Recently, technologies have been developed that offer the possibility of using algal biomass as feedstocks to energy producing systems – in addition to oil-derived fuels (Bird et al., 2011, 2012). Growing native mixed microalgal consortia for biomass in association with geothermal resources has the potential to mitigate negative impacts of seasonally low temperatures on biomass production systems as well as mitigate some of the challenges associated with growing unialgal strains. We assessed community composition, growth rates, biomass, and neutral lipid production of microalgal consortia obtained from geothermal hot springs in the Great Basin/Nevada area that were cultured under different thermal and light conditions. Biomass production rates ranged from 39.0 to 344.1 mg C L^−1^ day^−1^. The neutral lipid production in these consortia with and without shifts to lower temperatures and additions of bicarbonate (both environmental parameters that have been shown to enhance neutral lipid production) ranged from 0 to 38.74 mg free fatty acids (FFA) and triacylglycerols (TAG) L^−1 ^day^−1^; the upper value was approximately 6% of the biomass produced. The higher lipid values were most likely due to the presence of *Achnanthidium* sp. Palmitic and stearic acids were the dominant free fatty acids. The S/U ratio (the saturated to unsaturated FA ratio) decreased for cultures shifted from their original temperature to 15°C. Biomass production was within the upper limits of those reported for individual strains, and production of neutral lipids was increased with secondary treatment. All results demonstrate a potential of culturing and manipulating resultant microalgal consortia for biomass-based energy production and perhaps even for biofuels.

## Introduction

Environmental and biological fluctuations (e.g., temperature, light levels, nutrient availability, pH, grazers, etc.) make the maintenance of unialgal strains as a feedstock for fuels/biofuels challenging (Sheehan et al., 1998; Brennan and Owende, 2010). During the aquatic species program (ASP) that conducted a myriad of activities aimed at producing algae for oil (Sheehan et al., 1998), biomass recycling was an example of a method employed for species maintenance. Even when such measures were employed, shifts in the dominant taxa often occurred (Weissman and Benemann, 1979; Sheehan et al., 1998). Some have henceforth concluded that for successful cultivation for fuels, the best approach would be to allow a native species to invade production ponds (Sheehan et al., 1998). However, these native contaminants would have to be maintained/manipulated to be oleaginous for fuels production.

Recently, additional technologies have been developed that offer the possibility of using algal biomass as feedstocks to other energy producing systems beyond oil-derived fuels (Bird et al., 2011, 2012). Such developments make algal biomass – beyond that which is oleaginous – possible feedstocks that could help in meeting energy demands.

It is apparent that for such biomass-to-energy pathways to be developed and realized, high productivity, high yield, and low-cost-low-maintenance systems will be required. High production and yields that are able to be maintained despite time-varying conditions in temperatures and irradiances (that can vary on time scales of minutes to seasons) will be highly desirable.

Growing algal biomass in association with geothermal resources has the potential to mitigate negative impacts of low temperatures on biomass production systems – and thus, has the potential for maintaining production in areas where low seasonal temperatures might otherwise preclude high production. Moreover, cultivating microalgae in high-temperature environments has the potential to increase intrinsic growth rates and productivity. Throughout much of the arid west/southwest geothermal resources are abundant (Faulds et al., 2004) and are being further developed as a source of energy (United States Congress Senate Committee On et al., 1984). Moreover, the arid west/southwest is a location where irradiances are favorable for algal growth throughout much of the year (Davis, 2012). If water downstream from these power plants – or even directly from hot springs or wells – were to be used to heat algal productions systems (either directly or indirectly), the likelihood that such systems might become viable, for use in some fashion in the algal production industry, would be expanded.

To help in the overall evaluation of the potential for growing algal biomass in high productivity systems at moderately high temperatures, we cultivated mixed consortia from two hot springs in Nevada, evaluated their growth at moderately high varying temperatures and then evaluated potential manipulations that could possibly increase their oleaginous production as well. Results are evaluated in context of evaluating the potential of long-term maintenance of highly productive consortia as feedstock for energy.

## Materials and Methods

### Sample collection

Samples were collected for culturing from two geothermal hot spring sites: (1) Hazen (also known as Patua), located in central Nevada near Hwy 50 between Fernley and Fallon (39°35′ 57.0″ N, −119°6′ 40.0″ W) on 5/19/2011 and (2) Monitor (also known as Potts), located in central Nevada approximately 45 miles southeast of Austin (39°04′ 43.3″ N, −116°38′ 24.3″ W) on 10/29/2011. A benthic algal and sediment sample was collected at (1) Hazen at a temperature of 38°C and pH of 7.28 and (2) Monitor at a temperature of 41°C and pH of 6.87.

Algae and nutrient samples were collected for culturing and water chemistry. Algae samples were inoculated into 3N media prepared with 0.2 μm capsule filtered geothermal water from each site (here after referred to as Geo3). Water samples, for chemical analysis, were filtered *in situ* using a 0.4-μm pore polycarbonate filter and then frozen immediately until analysis. Water samples were analyzed using (1) a Lachat QuikChem FIA+ 8000 series for soluble reactive phosphorus (ortho-P), ammonium $\left({\mathrm{NH}}_{4}^{+}\right),$ silicon oxide (SiOx), and total combined ${\mathrm{NO}}_{2}^{-}$ and ${\mathrm{NO}}_{3}^{-}$ [ortho-P, 10-115-01-1-M (Liao, 2002); ${\mathrm{NH}}_{4}^{+},$ 10-107-06-2-C (Prokopy, 2003); SiOx, 31-114-27-1-D (Wolters, 2002); NOx, 10-107-04-1-C (Pritzlaff, 2000)] and (2) a Dionex ICS-1500 ion chromatograph for the anions and cations [fluoride (Fl^−^), chloride (Cl^−^), bromide (Br^−^), sulfate $\left({\mathrm{SO}}_{4}^{2-}\right),$ lithium (Li^+^), sodium (Na^+^), potassium (K^+^), magnesium (Mg^2+^), and calcium (Ca^2+^)]. Determination of anions and cations was performed using an IonPac^®^ analytical separatory column, guard columns (anions, AS14A and AG14A; cations, CS12A and CG12A), eluents (anions, sodium carbonate, and sodium biocarbonate; cations, methane sulfanic acid), and Dionex standards (anions, Seven Anion Standard II; cations, Six Anion Standard II).

### Initial mixed culture maintenance and experimental initiation

Consortia (250 mL) were used to inoculate 750 mL of media in 2 L baffled culture flasks. The cultures were maintained at 30°C (Hazen) or 40°C (Monitor) initially under continuous irradiance of 200 μE m^−2^ s^−1^, then transfered to a light:dark cycle of 12:12 hours, before being transferred to natural lighting within temperature controlled enclosures (Ecopods) in a green house setting.

### Mixed culture semi-continuous phase and incubation variance

The cultures were maintained in batch mode until they reached later stages of logarithmic growth, when they were then switched to semi-continuous cultures. The volumes for the dilutions were determined by the apparent growth rates using *in vivo* fluorescence (Fo) measures. Fo measures were performed using a Spectromax Gemini EM 96-well plate spectrofluorometer (excitation 440 nm, emission 680 nm). During the semi-continuous phase, samples were collected and cultures were diluted with fresh media on a daily basis.

### Growth rates

Growth rates were determined, by Fo measures, over the time series when the culture was in the exponential growth phase using Eq. 1:

$$\mu =\left[\ln\left(Fo_{t2}\right)-\ln\left(Fo_{t1}\right)\right]∕\Delta t$$

where μ was the growth rate, ln(Fo_t2_) was the natural log of the Fo reading at the end of the determined growth phase, ln(Fo_t1_) was the natural log of the Fo reading at the beginning of the determined growth phase, and Δ*t* was the time interval. The upper and lower limits were determined by a 95% confidence interval on the natural log of the exponential growth phase. *P*-values were generated and all values were below the critical value 0.05.

**Figure 1:**
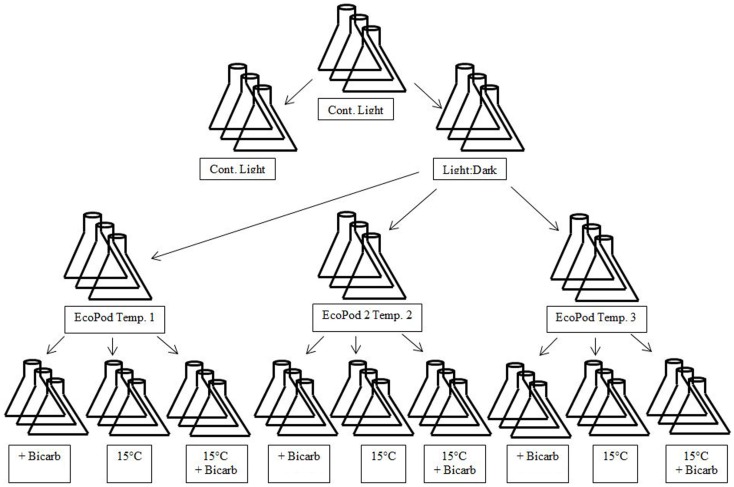
**Diagram of experimental design matrix**. EcoPods (incubators in a greenhouse) were maintained at three different temperatures for each culture – Hazen: 30 ± 3°C, 35 ± 2°C, and 40 ± 3°C and Monitor: 35 ± 2°C, 40 ± 3°C, and 45 ± 3°C. Secondary treatments consisted of the addition of bicarbonate, a shift to 15°C and a shift to 15°C with the addition of bicarbonate

The microalgal consortia sub-cultures were taken and transferred to three separate incubation conditions. The incubation conditions for Hazen samples were natural light in a greenhouse (EcoPod – EP) at (1) 30 ± 3°C, (2) 35 ± 2°C, and (3) 40 ± 3°C. The incubation conditions for Monitor samples were EP at (1) 35 ± 2°C, (2) 40 ± 3°C, and (3) 45 ± 3°C. After the cultures had acclimated to their respective conditions they were maintained in a semi-continuous state (Figure 1).

### Mixed culture secondary treatment

When a near steady-state was obtained, mixed cultures were combined and then split into 12 separate × 500 mL culture flasks for a given incubation condition. From the 12 replicates, 4 treatments (3 replicates per treatment) were achieved and each treatment was either: maintained at temperature, maintained at temperature with the addition of sodium bicarbonate (final concentration 4 mM), incubated at 15°C, or incubated at 15°C with the addition of sodium bicarbonate (final concentration 4 mM) – with the exception of the 40°C Hazen cultures that only underwent the temperature shift and the temperature shift with the addition of bicarbonate.

All mixed cultures were incubated for a 5-day period. Samples were taken daily for microscopy (fixed with glutaraldehyde 0.5% final concentration), measures of Fo, and stained with Nile Red (NR) for quantification of neutral lipids (15 μL of NR solution was added to 1 mL algal suspension, vortexed and 300 μL loaded into wells of a 96-well plate; fluorescence intensity was measured using a Spectromax Gemini EM 96-well plate spectrofluorometer: excitation 530 nm and emission 575 nm). Samples for measures of water chemistry, triacylglycerides (TAG), free fatty acids (FFA), and ash free dry weight (AFDW) were taken at the beginning and end of the 5-day period. AFDW samples were vacuum filtered onto pre-combusted (500°C for 1 h) GF/F filters, dried, and stored with desiccant. AFDW was then determined gravimetrically using standard methods (Clesceri et al., 1998). TAG and FFA samples were analyzed using electrospray tandem mass spectrometry (ESI-MS/MS) and ultra-performance liquid chromatography-tandem mass spectrometric (UPLC/MS) methods (Samburova et al., 2013).

### Biomass, TAG, and FFA determinations

Biomass, TAG, and FFA production rates were determined using the ADFW data. Biomass productivity was determined by AFDW at the beginning and end of the growth phase over the time of the growth phase (estimates of carbon production were made using an average C:AFDW ratio of 0.5g:g). The concentration of TAG and FFA (determined by ESI-MS/MS and UPLC/MS methods) for each sample was divided by the AFDW, for that sample, to determine the total TAG and FFA content per unit biomass.

## Results

### Chemical composition of geothermal waters

The chemical composition of the geothermal hot spring waters – Hazen and Monitor – was different; the majority of cation and anion concentrations in Hazen water were considerably higher (Table 1). Sodium was over 13 times higher in Hazen than Monitor water. Cations: lithium, potassium, and calcium were also higher in Hazen water, except magnesium, which was almost four times higher in Monitor water. Silica, nitrate plus nitrite, and phosphorous were higher in Hazen, as well.

**Table 1:** **Chemical composition of geothermal hot springs water from Hazen and Monitor hot springs**.

Site	Ortho-P (μM)	NH_4_ (μM)	SiOx (μM)	NO_2_ + NO_3_ (μM)	Li^+^ (μM)	Na^+^ (μM)	K^+^ (μM)	Mg^2+^ (μM)	Ca^2+^ (μM)	Fl^−^ (μM)	Cl^−^ (μM)	Br^−^ (μM)	${\mathrm{SO}}_{4}^{2-}$ (μM)
Hazen	1.29	14.55	1560.14	0.61	260.93	28248.66	989.30	26.98	1789.47	247.23	25163.14	36.13	3751.43
Monitor	0.31	13.87	468.91	n.d.	33.08	2082.41	329.22	97.18	1357.61	79.34	314.06	n.d.	560.00

### Culture experiments – EcoPods and secondary treatment

#### Community composition

The dominant taxa of the Hazen geothermal consortia, at 30, 35, and 40°C, were similar and consisted of *Achnanthidium* sp., *Aphanocapsa* sp., *Synechocystis* sp., and *Leptolyngbya* sp (Tables 2–4). The algal assemblage at the beginning and end of the treatment period was consistent for replicates of a specific treatment. Shifts in the dominant algal genus in the consortia, were seen at 30°C with the addition of bicarbonate, at 35°C, and at 35°C with the addition of bicarbonate. Dominance in the assemblages shifted from *Achnanthidium* sp. to either *Synechocystis* sp. or *Leptolyngbya* sp.

**Table 2:** **Relative percentage of dominate taxa by biovolume for the EcoPod (natural light) cultures at 30°C before and after secondary treatment; *N* = 3 for all counts, ±1 SD**.

	EP30	EP30	EP30/30	EP30/30 + B	EP30/15	EP30/15 + B
	6/28	8/22	8/27	8/27	8/27	8/27
*Leptolyngbya*	0 ± 0	5 ± 1	3 ± 0	20 ± 3	9 ± 1	8 ± 1
*Aphanocapsa*	26 ± 6	22 ± 4	24 ± 7	10 ± 1	29 ± 2	27 ± 2
*Synechocystis*	34 ± 11	34 ± 16	43 ± 17	52 ± 4	53 ± 11	46 ± 18
*Achnanthidium*	34 ± 12	25 ± 13	22 ± 13	1 ± 1	4 ± 4	15 ± 13
*Chroococcus*	6 ± 5	15 ± 3	7 ± 5	18 ± 10	5 ± 5	5 ± 7
*Synechococcus*	0 ± 0	0 ± 0	0 ± 0	0 ± 0	0 ± 0	1 ± 1

**Table 3:** **Relative percentage of dominate taxa by biovolume for the EcoPod (natural light) cultures at 35°C before and after secondary treatment; *N* = 3 for all counts, ±1 SD**.

	EP35	EP35	EP35/35	EP35/35 + B	EP35/15	EP35/15 + B
	7/18	8/22	8/27	8/27	8/27	8/27
*Leptolyngbya*	0 ± 0	13 ± 3	44 ± 13	16 ± 5	29 ± 4	20 ± 9
*Aphanocapsa*	53 ± 24	56 ± 8	35 ± 9	29 ± 2	40 ± 11	53 ± 5
*Synechocystis*	24 ± 21	15 ± 3	13 ± 3	51 ± 10	0 ± 0	0 ± 0
*Achnanthidium*	18 ± 18	16 ± 3	8 ± 2	4 ± 1	31 ± 2	27 ± 7
*Chroococcus*	0 ± 0	0 ± 0	0 ± 0	0 ± 0	0 ± 0	0 ± 0
*Synechococcus*	4 ± 5	0 ± 0	0 ± 0	0 ± 0	0 ± 0	0 ± 0

**Table 4:** **Relative percentage of dominate taxa by biovolume for the EcoPod (natural light) cultures at 40°C before and after secondary treatment; *N* = 3 for all counts, ±1 SD**.

	EP40	EP40	EP40/15	EP40/15 + B
	7/28	8/22	8/27	8/27
*Leptolyngbya*	0 ± 0	19 ± 5	7 ± 1	9 ± 1
*Aphanocapsa*	21 ± 5	39 ± 15	31 ± 5	52 ± 12
*Synechocystis*	45 ± 14	11 ± 4	61 ± 25	38 ± 6
*Achnanthidium*	21 ± 20	2 ± 2	0 ± 0	0 ± 0
*Chroococcus*	12 ± 21	0 ± 0	0 ± 0	0 ± 0
*Synechococcus*	1 ± 1	29 ± 11	1 ± 2	2 ± 2

Dominant taxa in the Monitor geothermal consortia, at 35, 40, and 45°C, were similar and consisted of *Oscillatorian* sp., *Synechocystis* sp., and *Leptolyngbya* sp. at the beginning of the secondary treatment phase. The algal assemblage at the beginning and end of the treatment period were consistent for replicates of a specific treatment. Shifts in the dominant genus in the consortia were seen at 45°C with and without the addition of bicarbonate to *Leptolyngbya* dominated assemblages.

#### Specific growth rates

##### Hazen

**Figure 2:**
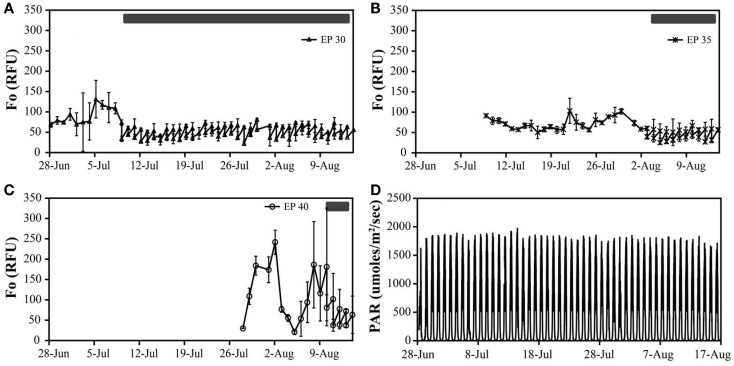
**The *in vivo* fluorescence (relative fluorescence units – RFU) of Hazen geothermal consortia at different incubation conditions**: EcoPod at 30°C (EP 30) with dilution rates of 60% from July 9th through August 14th **(A)**, EcoPod at 35°C (EP 35) with dilution rates of 50% from August 3rd through 14th **(B)**, EcoPod at 40°C (EP 40) with dilution rates of 45% from August 10th through 14th **(C)**. PAR measured in μmoles m^−2^ s^−1^ for the duration of the experiment **(D)**.

Cultures maintained at temperature with the addition of bicarbonate had higher growth rates (ranging from 0.63 to 0.79 doublings day^−1^) than those just maintained at temperature (ranging from 0.46 to 0.60 doublings day^−1^). The highest growth rate was observed in the cultures at 30°C with the addition of bicarbonate, averaging 0.79 doublings day^−1^. The temperature shift to 15°C decreased the growth rates in all cultures, and in some cases to a growth rate of 0 (Figure 2; Table 5).

**Table 5:** **Growth rates, triacylglycerides (TAG), and free fatty acids (FFA), biomass production rates of Hazen and Monitor consortia incubated at different temperatures with and without the addition of bicarbonate**.

Initial temp/secondary treatment	Hazen growth rate (doublings day^−1^) *N* = 3, ±1 SD	Monitor growth rate (doublings day^−1^) *N* = 3, ±1 SD	Hazen estimated biomass production rates (mg C L^−1 ^day^−1^)	Monitor estimated biomass production rates (mg C L^−1^ day^−1^)	Hazen estimated TAG and FFA production rates (mg L^−1 ^day^−1^)	Monitor estimated TAG and FFA production rates (mg L^−1^ day^−1^)
30/30	0.60 ± 0.24	n.a.	129.7	n.a.	2.82	n.a.
30/30 + B	0.79 ± 0.48	n.a.	344.1	n.a.	17.80	n.a.
30/15	0.35 ± 0.15	n.a.	0.00	n.a.	38.74	n.a.
30/15 + B	0.22 ± 0.09	n.a.	0.00	n.a.	38.52	n.a.
35/35	0.46 ± 0.09	0.40 ± 0.20	119.0	123.4	0.95	n.a.
35/35 + B	0.63 ± 0.10	0.53 ± 0.05	309.0	266.3	16.06	n.a.
35/15	0.07 ± 0.02	0.13 ± 0.11	0.00	0.00	21.61	n.a.
35/15 + B	0.07 ± 0.03	0.00 ± 0.19	0.00	0.00	31.03	n.a.
40/40	n.a.	0.50 ± 0.28	n.a.	140.0	n.a.	3.34
40/40 + B	n.a.	0.62 ± 0.21	n.a.	382.4	n.a.	24.65
40/15	0.02 ± 0.02	0.00 ± 0.15	0.00	0.00	0.20	16.82
40/15 + B	0.10 ± 0.06	0.00 ± 0.17	0.00	0.00	n.d.	10.67
45/45	n.a.	0.21 ± 0.19	n.a.	39.0	n.a.	n.a.
45/45 + B	n.a.	0.29 ± 0.17	n.a.	46.5	n.a.	n.a.
45/15	n.a.	0.00 ± 0.03	n.a.	0.00	n.a.	n.a.
45/15 + B	n.a.	0.00 ± 0.05	n.a.	0.00	n.a.	n.a.

*n.a., not applicable; n.d., not detected*.

##### Monitor

**Figure 3:**
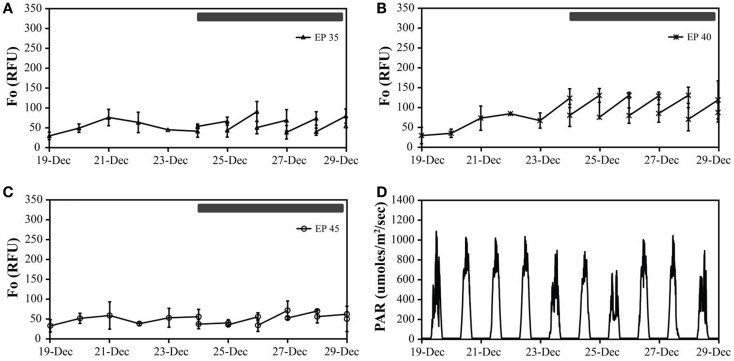
**The *in vivo* fluorescence of Monitor geothermal consortia at different incubation conditions**: EcoPod at 35°C (EP 35) with dilution rates of 40% from December 24th through 29th **(A)**, EcoPod at 40°C (EP 40) with dilution rates of 50% from December 24th through 29th **(B)**, EcoPod at 45°C (EP 45) with dilution rates of 20% from December 24th through 29th **(C)**. PAR measured in μmoles m^−2^ s^−1^ for the duration of the experiment **(D)**.

Cultures maintained at temperature with the addition of bicarbonate had higher growth rates (ranging from 0.29 to 0.62 doublings day^−1^) than those just maintained at temperature (ranging from 0.21 to 0.50 doublings day^−1^). The highest growth rate was achieved by the cultures at 40°C with the addition of bicarbonate, averaging 0.62 doublings day^−1^. The temperature shift to 15°C decreased the growth rates in all cultures, and in some case to a growth rate of 0 (Figure 3; Table 5).

#### Biomass productivity

##### Hazen

Biomass productivity followed the same trends as the measured growth rates. The cultures maintained at 30°C with the addition of bicarbonate produced the greatest biomass – 344.1 mg C L^−1^ day^−1^. The culture at 35°C had the lowest biomass production – 118.9 mg C L^−1^ day^−1^. The cultures maintained at temperature (excluding 45°C) – with the addition of bicarbonate – had an average percent increase in biomass production of 163% more than the cultures that did not have bicarbonate additions (Table 5).

##### Monitor

Biomass productivity also followed the same trends as the measured growth rates. The cultures maintained at 40°C with the addition of bicarbonate produced the greatest biomass – 382.3 mg C L^−1^ day^−1^. The culture at 45°C had the lowest biomass production – 39.0 mg C L^−1^ day^−1^. The cultures maintained at temperature – with the addition of bicarbonate – had an average percent increase in biomass production of 102% more than the cultures that did not have bicarbonate additions (Table 5).

#### Nile Red, FFA, and TAGs productivity and composition

**Figure 4:**
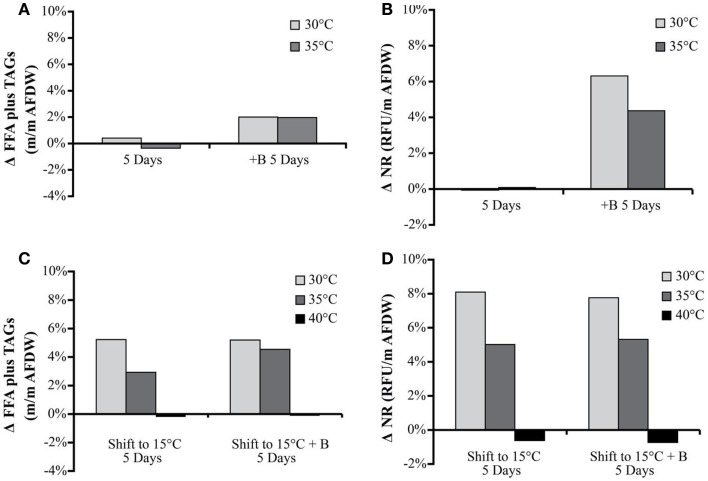
**Hazen geothermal consortia: change of FFA and TAG (%, w/w) after 5 days, with and without the addition of bicarbonate (A), change of NR after 5 days with and without the addition of bicarbonate (B), as well as a shift to 15°C from their original incubation conditions with and without the addition of bicarbonate (C,D)**.

The NR values showed an average increase in neutral lipid accumulation per unit biomass of only 6–8% with secondary treatments (except 40°C, which actually showed a decrease) (Figure 4). The change in Nile Red values in the biomass was similar to the trends observed in TAG and FFA accumulation.

The FFA and TAG concentration per amount of biomass, for the Hazen cultures, marginally increased with the addition of bicarbonate – from approximately 1–3%. The addition of bicarbonate, for cultures maintained at temperature, increased the estimated FFA and TAG productivity from (1) 2.82 to 17.8 mg L^−1^ day^−1^ for 30°C and (2) 0.95 to 16.06 mg L^−1^ day^−1^ for 35°C (Table 5). The amount of FFA and TAG per amount biomass increased with decreasing temperature by 3–5% – except in the 40°C cultures (Figure 4). A decrease in temperature increased the estimated FFA and TAG productivity, to 21.61–38.74 mg L^−1^ day^−1^. The Monitor cultures showed an increase in TAG and FFA per amount biomass with both the addition of bicarbonate and a temperature drop (from 3.34 mg L^−1^ day^−1^ to a maximum value of 24.65 mg L^−1^ day^−1^).

FFA and TAG profiles were analyzed for the following treatment conditions: (1) maintained at temperature, (2) maintained at temperature with the addition of bicarbonate, (3) shifted to 15°C, and (4) shifted to 15°C with the addition of bicarbonate. FFA composition stayed relatively constant with both the addition of bicarbonate and with the temperature shift (Table 6). The TAG diversity increased after the fifth day of the incubation period in all cultures. A shift was seen in the TAG composition to a decrease in chain length and increase in unsaturation at the cooler temperatures (Table 7).

**Table 6:** **The composition of FFA in Hazen geothermal consortia (%, w/w) at 0 and 5 days, with and without the addition of bicarbonate (A) as well as a shift to 15°C from their original incubation conditions, with and without the addition of bicarbonate (B)**.

Free fatty acid	0 Days			5 Days			5 Days + Bicarb		
	30°C		35°C	30°C		35°C	30°C		35°C
**(A)**									
Linolenic acid C18:3	0.5		0.4	1.4		1.0	n.d.		1.1
Linoleic acid C18:2	1.6		2.9	5.0		2.9	0.6		2.7
Palmitic acid C16:0	29.7		26.5	14.6		20.3	29.6		18.6
Palmitoleic acid C16:1	17.7		19.6	37.6		28.4	15.1		40.8
Oleic acid C18:1	4.7		5.7	9.3		8.7	3.9		8.0
Hexadecatrienoic acid C16:3	n.d.		n.d.	0.1		n.d.	n.d.		n.d.
Hexadecadienoic acid C16:2	1.6		1.6	6.4		3.2	n.d.		0.6
Stearic acid C18:0	42.7		36.3	11.6		21.9	50.3		17.0
Arachidonic acid C20:4	1.6		6.5	13.2		13.2	0.6		11.1
Eicosadienoic acid C20:2	n.d.		0.4	0.5		0.3	n.d.		n.d.
Eicosenoic acid C20:1	n.d.		n.d.	0.2		n.d.	n.d.		n.d.

**Free fatty acid**				**15°C Shift**			**15°C Shift**		
	**0 Days**			**5 Days**			**5 Days + Bicarb**		
	**30°C**	**35°C**	**40°C**	**30°C**	**35°C**	**40°C**	**30°C**	**35°C**	**40°C**

**(B)**									
Linolenic acid C18:3	0.5	0.4	n.d.	5.9	1.9	n.d.	5.7	5.2	n.d.
Linoleic acid C18:2	1.6	2.9	1.6	5.5	2.5	10.1	4.6	6.4	11.4
Palmitic acid C16:0	29.7	26.5	32.4	12.8	17.3	26.8	12.5	13.1	24.0
Palmitoleic acid C16:1	17.7	19.6	7.6	39.8	48.6	6.1	40.8	38.0	15.3
Oleic acid C18:1	4.7	5.7	5.9	7.3	3.4	14.5	7.3	7.1	13.1
Hexadecatrienoic acid C16:3	n.d.	n.d.	n.d.	0.4	0.1	n.d.	0.4	0.3	n.d.
Hexadecadienoic acid C16:2	1.6	1.6	n.d.	9.2	4.3	n.d.	9.4	9.0	n.d.
Stearic acid C18:0	42.7	36.3	52.4	5.9	16.8	42.5	7.9	10.1	36.2
Arachidonic acid C20:4	1.6	6.5	n.d.	12.8	4.8	n.d.	11.1	10.3	n.d.
Eicosadienoic acid C20:2	n.d.	0.4	n.d.	0.4	0.2	n.d.	0.2	0.4	n.d.
Eicosenoic acid C20:1	n.d.	n.d.	n.d.	0.1	0.1	n.d.	0.1	0.1	n.d.

*n.d., limit of detection (0.03 μg mL^−1^)*.

**Table 7:** **The composition of TAG in Hazen geothermal consortia (%, w/w) at 0 and 5 days, with and without the addition of bicarbonate (A) as well as a shift to 15°C from their original incubation conditions, with and without the addition of bicarbonate (B)**.

TAG	0 Days			5 Days			5 Days + Bicarb		
	30°C		35°C	30°C		35°C	30°C		35°C
**(A)**									
C16:1/C16:1/C16:1	n.d.		n.d.	10.8		10.0	16.9		10.4
C16:0/C16:1/C16:1	n.d.		25.0	35.0		34.6	34.1		34.5
C16:0/C16:0/C16:1	n.d.		25.0	26.8		28.5	22.9		24.7
C16:0/C16:0/C16:0	n.d.		n.d.	0.6		0.8	0.3		0.6
C18:3/C16:1/C16:1	n.d.		n.d.	1.3		0.8	0.7		0.6
C18:3/C16:1/C16:0	n.d.		n.d.	1.9		2.3	1.7		1.7
C18:3/C16:0/C16:0	n.d.		n.d.	3.2		3.8	4.1		3.4
C18:1/C16:1/C16:0	n.d.		n.d.	5.1		5.4	6.9		6.3
C18:1/C16:0/C16:0	n.d.		n.d.	3.2		3.1	3.3		3.1
C18:0/C16:0/C16:0	n.d.		n.d.	n.d.		n.d.	0.1		0.1
C18:3/C18:1/C16:1	n.d.		n.d.	5.1		4.6	5.4		8.8
C18:3/C18:1/C16:0	n.d.		n.d.	2.5		2.3	1.4		3.4
C18:1/C18:1/C16:1	n.d.		n.d.	1.3		0.8	0.7		1.2
C18:1/C18:1/C16:0	n.d.		n.d.	0.6		0.8	0.3		0.3
C18:1/C18:0/C16:0	n.d.		n.d.	0.6		n.d.	0.2		0.2
C18:0/C18:0/C16:0	n.d.		n.d.	n.d.		n.d.	n.d.		n.d.
C18:0/C18:1/C18:3	n.d.		n.d.	n.d.		n.d.	0.2		0.2
C18:1/C18:1/C18:1	n.d.		n.d.	0.6		0.8	0.1		0.2
C18:0/C18:1/C18:1	n.d.		n.d.	n.d.		n.d.	0.1		0.1
C18:0/C18:0/C18:1	100.0		50.0	1.3		1.5	0.3		0.1
C20:4/C18:1/C16:2	n.d.		n.d.	n.d.		n.d.	0.3		0.2

**TAG**				**15°C Shift**			**15°C Shift**		
	**0 Days**			**5 Days**			**5 Days + Bicarb**		
	**30°C**	**35°C**	**40°C**	**30°C**	**35°C**	**40°C**	**30°C**	**35°C**	**40°C**

**(B)**									
C16:1/C16:1/C16:1	n.d.	n.d.	n.d.	20.8	14.4	n.d.	18.5	18.8	n.d.
C16:0/C16:1/C16:1	n.d.	25.0	n.d.	27.9	39.0	n.d.	34.3	37.1	n.d.
C16:0/C16:0/C16:1	n.d.	25.0	n.d.	19.3	22.5	n.d.	19.6	19.8	n.d.
C16:0/C16:0/C16:0	n.d.	n.d.	n.d.	0.1	0.1	n.d.	0.1	0.1	n.d.
C18:3/C16:1/C16:1	n.d.	n.d.	n.d.	1.7	0.7	n.d.	1.0	0.9	n.d.
C18:3/C16:1/C16:0	n.d.	n.d.	n.d.	6.2	4.1	n.d.	3.8	4.0	n.d.
C18:3/C16:0/C16:0	n.d.	n.d.	n.d.	6.5	5.6	n.d.	6.7	7.2	n.d.
C18:1/C16:1/C16:0	n.d.	n.d.	n.d.	7.3	7.3	n.d.	8.8	8.7	n.d.
C18:1/C16:0/C16:0	n.d.	n.d.	n.d.	2.2	1.8	n.d.	1.8	1.4	20.0
C18:0/C16:0/C16:0	n.d.	n.d.	n.d.	n.d.	n.d.	n.d.	n.d.	n.d.	n.d.
C18:3/C18:1/C16:1	n.d.	n.d.	n.d.	4.6	2.3	n.d.	3.2	0.1	n.d.
C18:3/C18:1/C16:0	n.d.	n.d.	n.d.	1.3	0.7	n.d.	0.7	0.5	20.0
C18:1/C18:1/C16:1	n.d.	n.d.	n.d.	1.0	0.6	n.d.	0.6	0.6	20.0
C18:1/C18:1/C16:0	n.d.	n.d.	n.d.	0.4	0.3	n.d.	0.3	0.3	20.0
C18:1/C18:0/C16:0	n.d.	n.d.	n.d.	0.1	0.1	n.d.	0.1	0.1	n.d.
C18:0/C18:0/C16:0	n.d.	n.d.	n.d.	n.d.	n.d.	n.d.	n.d.	n.d.	n.d.
C18:0/C18:1/C18:3	n.d.	n.d.	n.d.	0.1	0.1	n.d.	0.1	0.1	n.d.
C18:1/C18:1/C18:1	n.d.	n.d.	n.d.	0.1	0.1	n.d.	0.1	n.d.	20.0
C18:0/C18:1/C18:1	n.d.	n.d.	n.d.	0.1	0.1	n.d.	n.d.	n.d.	n.d.
C18:0/C18:0/C18:1	100	50.0	n.d.	n.d.	n.d.	100	n.d.	0.1	n.d.
C20:4/C18:1/C16:2	n.d.	n.d.	n.d.	0.4	0.2	n.d.	0.3	0.2	n.d.

*n.d., limit of detection (0.03 μg mL^−1^)*.

## Discussion

Owing to the potential for energy production, it is imperative to determine algal consortia production rate of biomass and lipids, from high-temperature environments under culturing conditions. Cultivation conditions, such as temperatures and secondary treatment, can maximize production rates of algal biomass or increase neutral lipid concentrations. Microalgal consortia collected from naturally high-temperature environments present the possibility of robust communities, potentially having the advantage of resistance to invasion in production systems, and for elevated growth rates – therefore, biomass. Growing algae at higher temperature requires more energy to maintain; however, coupling this system with a geothermal resource would reduce energy requirements for heating and have the added bonus of consistent temperatures in all seasons. High biomass production rate were obtained from geothermal microalgal consortia.

Biomass production rate were similar for both Hazen and Monitor cultures over the range of temperature treatments (except Monitor at 45°C, which had <50% the production rate of the other cultures). Microalgae have evolved so that their optimum temperature is the same as their environmental temperature (Brock, 1967), which would explain why increasing the incubation temperature did not increase biomass production rates.

Biomass production rates (39.0–344.1 mg C L^−1^ day^−1^) achieved by geothermal microalgal consortia (Hazen/Monitor) were comparable to those reported for individual strains. The reported biomass production rates of various strains (taxa and incubation temperature: *Nannochloropsis* sp. 28 ± 1°C, *Isochrysis* sp. 22 ± 2°C, *Tetraselmis* sp. 28 ± 1°C, *Neochloris oleoabundans* 25.6°C, *Chlorella* sp. 23–29°C, *Dunaliella salina* 23–29°C, and *Chlorella vulgaris* 23–29°C) range from 42.3 to 7100 mg C L^−1^ day^−1^ (Huerlimann et al., 2010; Araujo et al., 2011; Kim, 2011; Zhou et al., 2011; Murray et al., 2012). The incubation temperatures for the strains reported in the literature were mainly below those that were used in this experiment. The production rates, with the addition of bicarbonate, were enhanced. The addition of bicarbonate allows for enhanced inorganic carbon uptake to produce cellular material and thereby achieving maximum productivity. The poor solubility of CO_2_ in water (1.25 g kg^−1^ water at 30°C and 1 atm) can lead to growth inhibition due to carbon limitations (Smith and Bidwell, 1989; Giordano et al., 2005; Aishvarya et al., 2012). CO_2_ solubility decreases with increasing temperature and at higher temperature ranges there can be severe loss of gaseous CO_2_ in aquatic systems (Dodds et al., 1956; Carroll et al., 1991). Given what is known about CO_2_ solubility it is logical to propose that geothermal systems might be carbon limited (due to being at elevated temperatures); the demonstrated increase in biomass production with the addition of bicarbonate further supports this hypothesis. The result of higher biomass production validates the idea that biomass yields can be increased with the utilization of native geothermal consortia.

Biomass production ranged from approximately 3.8–34.5 g C m^−2^ day^−1^ with the addition of bicarbonate. The long-term industry standard, set by U.S. Department of Energy’s Aquatic Species Program, for biomass production from microalgae is 50 g dry weight m^−2^ day^−1^ to be cost effective and competitive with fossil fuels (Sheehan et al., 1998). Production rates were converted from a volume basis (L) to an area production bases (m^2^) assuming that volumetric bases can be maintained in a raceway pond at a height of 30 cm – optimal depth for an open-race way pond for comparison to the industrial standard (Sheehan et al., 1998). The majority of open-race way ponds range from 10 to 50 cm in depth. The shallow depths used in cultivation have several advantages: (1) effective utilization of mixing energy, (2) less concentration needed at harvesting, and (3) dense cultures maintain photosynthetic efficiency because at deeper depths light becomes attenuated due to self-shading (Terry and Raymond, 1985; Sheehan et al., 1998; Brennan and Owende, 2010; Stephenson et al., 2011). However, the production rates are based on the experimental culture conditions (2 L culture flasks with a media height of 10 cm) and it remains to be seen how the cultures would perform on a larger scale within a pond or bioreactor.

The neutral lipid production of the cultures showed variations based on treatment. The FFA and TAG production from geothermal microalgal consortia, with and without secondary treatment, ranged from 0 to 38.74 mg L^−1^ day^−1^. Zhou et al. (2011) investigated the lipid productivity of 17 strains, mainly *Chlorella* sp. (incubated at unknown temperature) and reported values ranging between 0.0369 and 94.8 mg L^−1^ day^−1^, with an average lipid productivity of 54.97 mg L^−1^ day^−1^. Mata et al. (2010) reported values of lipid productivity for 29 strains that ranged from 10.3 to 142.0 mg L^−1^ day^−1^. Lipid production from the microalgal consortia was affected by the addition of bicarbonate and for some cultures by a temperature drop, with the exception of the 40°C cultures. The addition of bicarbonate in the lower temperature experiment (that came from the 30°C cultures), did not increase TAG and FFA accumulation. This lack of accumulation could have been due to increased CO_2_ solubility at lower temperatures rendering the additional carbon source unnecessary. In the 40°C culture the lower temperature experiment (with and without the addition of bicarbonate) showed reduced TAG and FFA content; this could have been due to general loss in culture biomass. Adequate dissolved inorganic carbon must be present for new lipid synthesis from carbon fixation. Inorganic carbon (in the form of bicarbonate) was supplied because it is an effective lipid accumulation trigger that induces carbon storage metabolic activity (Gardner et al., 2012, 2013; White et al., 2013). Although the addition of bicarbonate increased lipid productivity, values were still in the lower range of those reported for algal strains. Unfavorable growth conditions can cause a cessation of cellular replication and thereby also increasing the accumulation of FFA and TAG (Renaud et al., 1991; Sheehan et al., 1998; Widjaja et al., 2009). The highest values of FFA and TAG production were seen in the Hazen cultures, with a shift to 15°C with and without the addition of bicarbonate, and were approximately 6% of the biomass produced. The neutral lipid production in these cultures was most likely due to the presence of *Achnanthidium* sp.; however, these values were still below the average of the reported values for oleaginous strains.

The composition of FFA and TAG were also affected by the temperature decrease. FFA and TAG compositions followed the general trend of increasing unsaturation with decreasing temperature (Hu et al., 2008). The S/U ratio (the saturated to unsaturated FA ratio) decreased for cultures shifted from their original temperature to 15°C: (1) from 2.62 to 0.22 at 30°C, (2) from 1.69 to 0.41 at 35°C, and (3) from 5.61 to 1.89 at 40°C. Cultures from both 30 and 35°C, when shifted to 15°C, increased in palmitoleic acid (C16:1) content. A desaturation of palmitic acid to palmitoleic acid has been seen in *Anabaena variabilis* (Sato and Murata, 1980). The trend toward a decrease in chain length and an increase in unsaturation is based on maintaining membrane fluidity at cooler temperatures (Cronan and Gelmann, 1975; McElhaney and Souza, 1976; Hochachka and Somero, 1984). Triacylglycerides diversity increased by the end of the incubation period in all cultures and the trend of decreased chain length and increased unsaturation was seen at cooler temperatures.

The degree of unsaturation and common chain length of the FFAs found in the geothermal microalgal consortia were suitable for biodiesel production (Leonardi et al., 2011). The content of linolenic acid in all cultures – before and after secondary treatment – was below 12% (%, m/m), which is the maximum allowed by the European standard EN 14214. The most common chain length for polyunsaturated fatty acid (PUFA) was 16, with a maximum of 20.

The biomass production rates reported in this study show the potential of consortia as a source of alternative energy. Biomass production was within the upper limits of those reported for individual strains. Production of neutral lipids was increased with secondary treatment but still not comparable with oleaginous strains. Further work needs to be done on various high-temperature, microalgal consortia to establish the expected range for consortia production rates.

## Author Contributions

Kathryn F. Bywaters: conception of experimental design, performed experiments, post processed and analysed of data, and drafted the paper. Christian H. Fritsen: conception of experimental design, analysis of data, and drafted the paper.

## Conflict of Interest Statement

The Guest Associate Editor Umakanta Jena declares that, despite being affiliated to the same institution as authors, the review process was handled objectively and no conflict of interest exists. The authors declare that the research was conducted in the absence of any commercial or financial relationships that could be construed as a potential conflict of interest.
